# Genes Influencing Circadian Differences in Blood Pressure in Hypertensive Mice

**DOI:** 10.1371/journal.pone.0019203

**Published:** 2011-04-26

**Authors:** Francine Z. Marques, Anna E. Campain, Pamela J. Davern, Yee Hwa J. Yang, Geoffrey A. Head, Brian J. Morris

**Affiliations:** 1 Basic and Clinical Genomics Laboratory, School of Medical Sciences and Bosch Institute, The University of Sydney, Sydney, Australia; 2 School of Mathematics and Statistics, The University of Sydney, Sydney, Australia; 3 Neuropharmacology Laboratory, Baker IDI Heart Research Institute, Melbourne, Australia; University of Florida, United States of America

## Abstract

Essential hypertension is a common multifactorial heritable condition in which increased sympathetic outflow from the central nervous system is involved in the elevation in blood pressure (BP), as well as the exaggerated morning surge in BP that is a risk factor for myocardial infarction and stroke in hypertensive patients. The Schlager BPH/2J mouse is a genetic model of hypertension in which increased sympathetic outflow from the hypothalamus has an important etiological role in the elevation of BP. Schlager hypertensive mice exhibit a large variation in BP between the active and inactive periods of the day, and also show a morning surge in BP. To investigate the genes responsible for the circadian variation in BP in hypertension, hypothalamic tissue was collected from BPH/2J and normotensive BPN/3J mice at the ‘peak’ (n = 12) and ‘trough’ (n = 6) of diurnal BP. Using Affymetrix GeneChip® Mouse Gene 1.0 ST Arrays, validation by quantitative real-time PCR and a statistical method that adjusted for clock genes, we identified 212 hypothalamic genes whose expression differed between ‘peak’ and ‘trough’ BP in the hypertensive strain. These included genes with known roles in BP regulation, such as vasopressin, oxytocin and thyrotropin releasing hormone, as well as genes not recognized previously as regulators of BP, including chemokine (C-C motif) ligand 19, hypocretin and zinc finger and BTB domain containing 16. Gene ontology analysis showed an enrichment of terms for inflammatory response, mitochondrial proton-transporting ATP synthase complex, structural constituent of ribosome, amongst others. In conclusion, we have identified genes whose expression differs between the peak and trough of 24-hour circadian BP in BPH/2J mice, pointing to mechanisms responsible for diurnal variation in BP. The findings may assist in the elucidation of the mechanism for the morning surge in BP in essential hypertension.

## Introduction

Essential hypertension is a common [1] multifactorial condition involving the influence of numerous, mostly unidentified genes, generally thought to have small effects on blood pressure (BP) [2]. Essential hypertensive patients display an exaggerated increase in BP levels in the morning, referred to as the morning BP surge [3]. The basis of this phenomenon is not well understood. The morning surge is known to increase risk of cardiovascular events [4]. The identification of the mechanisms responsible for circadian variations in BP, particularly in hypertensive patients, should assist in the design of new strategies for resolving the pathophysiology of this condition.

**Figure 1:**
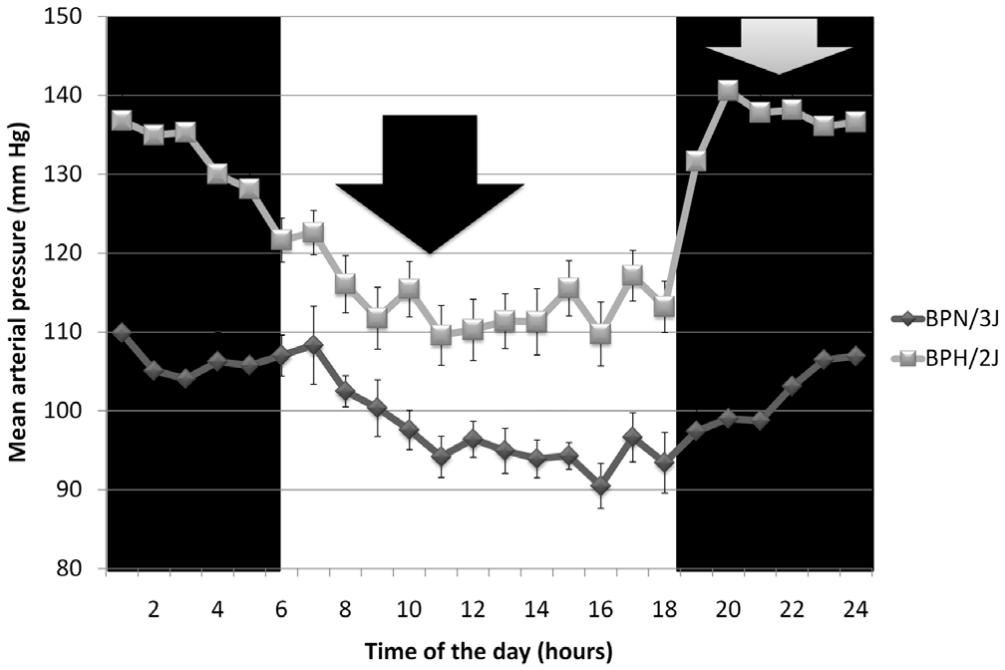
Circadian variation of blood pressure in the Schlager hypertensive and normotensive strains. Hourly averaged data showing the circadian variation of mean arterial pressure (mm Hg) during the active (night; outer black panels) and inactive (day; middle white panel) phases in 23 week-old BPN/3J and BPH/2J mice. Values are mean ± SEM for comparisons between strains across the entire 24 hours. Black arrow indicates when ‘trough’ BP samples were collected, and gray arrow indicates when ‘peak’ BP samples were collected. Adapted from Davern *et al.* [8].

In both animal models and humans, there is increasing evidence that the sympathetic nervous system (SNS) is involved in the development and progression of hypertension [5]. The causes of the sympathetic activation are, however, still unclear. The SNS is also a key regulator of the morning BP surge phenomenon [6], and the use of drugs which target the SNS are effective in reducing it [7]. It was reported that acute sympathetic blockade decreases BP in the Schlager BPH/2J hypertensive mouse strain [8], consistent with involvement of the SNS in this genetic model of hypertension. The hypertensive strain presents a very distinctive circadian variation of BP similar to humans with essential hypertension. During the active phase average mean arterial pressure (MAP) of the BPH/2J strain is 30 mm Hg higher than in the normotensive (BPN/3J) strain, and during the inactive phase is 16 mm Hg higher (Figure 1) [8]. Moreover, during the active phase, hypothalamic regions in the Schlager hypertensive mouse, specifically the paraventricular nucleus (PVN) and dorsomedial hypothalamus (DMH), exhibit higher neuronal activation than is seen in the BPN/3J [8]. Importantly, the PVN and DMH are brain regions known to be critical for the regulation of cardiovascular autonomic function [9], [10]. These hypothalamic regions are therefore likely to be important for the exaggerated circadian variation of BP in BPH/2J mice.

The aim of the present study was to identify, at the genome-wide level, the genes and imputed mechanisms in the hypothalamus that contribute to the higher BP in the active (dark phase) period in the Schlager hypertensive mouse. Although the hypothalamus is known to be a major regulator of the normal circadian rhythm and level of BP, our objective was not to identify clock genes associated with normal changes of BP. Therefore we used a statistical analysis which sought to eliminate clock genes by first comparing samples from BPH/2J to those for the control collected at the same time, i.e., prior to comparing hypertensive samples collected at ‘peak’ or ‘trough’ BP.

## Methods

### Ethics Statement

This study was approved by the Alfred Hospital Animal Ethical Review Committee (Permit number: E/0866/2009/B).

### Samples and tissue collection

Radiotelemetry studies by ourselves [8], [11], as well as tail-cuff measurements [12], have shown that BPH/2J hypertensive mice have high overall MAP of 127±2 mm Hg [8], while BPN/3J mice have normal overall MAP of 111±1 mm Hg [8]. Moreover, the hypertensive strain shows an exaggerated day-night difference (17±2 mm Hg) compared to the normotensive strain (6±1 mm Hg) and normal BP C57/B16 mice (8±2 mm Hg) [8].

In the present study, adult (19–26 week old) BPH/2J mice and age-matched BPN/2J mice (n = 3/group, ‘trough’ BP) were killed with an overdose of pentobarbitone (Lethobarb) in the inactive period, when the MAP levels of the BPH/2J and BPN/3J models differ by only 16 mm Hg [8]. BPH/2J mice and age-matched BPN/2J mice (n = 6/group, ‘peak’ BP) were killed in the same way at the peak of the circadian variation in BP, when average MAP difference between the strains was maximal (30 mm Hg) [8]. Figure 1 indicates the times when the mice were killed. The PVN and DMH, as defined by known anatomical boundaries [13], were removed immediately after death by PJD, who has extensive experience in dissecting PVN and DMH regions of the hypothalamus [8], [13]. The tissue was first preserved in dry ice and later transferred to a –80°C freezer and used for microarray experiments within 7 days. Each animal was considered an individual sample and no pooling was performed.

### RNA extraction and quality and quantity assessment

The RNeasy kit (Qiagen) was used for RNA extraction and was performed according to the manufacturer's recommendations. RNA quality was confirmed based on a RNA integrity number (RIN) higher than 8 by use of an electrophoresis bioanalyzer (2100 Agilent Bioanalyzer). This step was carried out by the Ramaciotti Centre for Gene Function Analysis, University of New South Wales, Sydney, Australia. Quantification involved spectrophotometry (NanoDrop® ND-100 spectrophotometer, Thermo Scientific) in the Laboratory at the University of Sydney.

### Microarray experiments and analyses

mRNA was converted to single-stranded DNA, labeled and hybridized to GeneChip® Mouse Gene 1.0 ST Arrays (Affymetrix), which analyze 28,869 gene transcripts using 764,885 probe sets (on average 27 probes per gene), all according to the manufacturer's instructions, and with the assistance of the Ramaciotti Centre. Samples were normalized using robust-multi-array analysis (RMA) [14]. The data set obtained has been deposited in the NCBI Gene Expression Omnibus database according to MIAME guidelines with series accession number GSE26007.

Direct comparison of differentially expressed genes between ‘trough’ and ‘peak’ BP samples would normally identify many “clock” genes that are of limited interest. Thus gene expression related to the circadian differences in hypertension were found by first adjusting for the circadian differences from BPN/3J. This analysis was performed using the adjusted fold difference (*aFD*) statistics we described previously [15]. Differentially expressed genes were selected based on an absolute *aFD* value exceeding 1.5, where positive *aFD* values indicate higher expression at ‘peak’ BP and negative *aFD* values indicate higher expression ‘trough’ BP in the Schlager hypertensive mouse. Hierarchical clustering using Euclidean distance was performed with TMeV 4.5 [16].

The Gene Ontology (GO) database [17] was used to further interpret the differentially expressed gene data set and to identify over-represented functional groups of genes. A hypergeometric test using *GOstats* [18] was used to determine if particular GO terms were more significantly over or under represented in the differentially expressed gene list than the gene list of the entire array. Up-regulated and down-regulated genes were examined separately. A gene set test (GST), implemented via the Limma package [19], was used to highlight pathways that are differentially expressed as a set, for all genes ranked via *P* values, and adjusted by false discovery rate (FDR). In both the GO and GST analyses, ontologies with an overall probe count of less than 5 were removed.

Using the ‘Core Analysis’ function in the Ingenuity Pathway Analysis (IPA, Ingenuity® Systems, www.ingenuity.com) application, molecular networks were built. Briefly, a data set containing differentially expressed genes and respective fold differences were uploaded into the application. These genes were then correlated based on previous association between genes or proteins and known functional roles of genes. The biological relationship between two genes, represented as nodes, is shown as a line. Nodes with different shapes indicate different functional class.

### Semi-quantitative real-time PCR (qPCR)

qPCR was conducted to confirm the results for genes whose functions were considered to be of possible interest in hypertension. The first-strand complementary synthesis reaction was performed using the SuperScript® VILO™ cDNA Synthesis Kit (Invitrogen). Amplification reactions used the EXPRESS SYBR® GreenER™ qPCR reagent system (Invitrogen) in a Light Cycler 480 qPCR machine (Roche). Primers were specifically designed around the most differentially expressed probe in the transcript cluster of each gene using Primer3 [20]. Where possible, primers were designed to flank an exon-exon junction. Primer and conditions used are indicated in Table 1. Samples were run in duplicate. The specificity of the qPCR was ensured by melting curve analysis and agarose gel electrophoresis (data not shown). The β-actin mRNA (*Actb*) was used as the reference gene. The comparative C_T_ statistical method was used to assess significance [21].

**Table 1:** Selected genes differentially expressed at ‘peak’ BP in the Schlager hypertensive mouse compared to ‘trough’ BP samples, showing primers, qPCR conditions and adjusted fold difference (*aFD*) values for both qPCR and microarray experiments.

Official gene symbol	GenBank Accession #	Primer Sequence (5′ → 3′)	Concentration	Annealing temperature	*aFD* value (qPCR)	*aFD* value (arrays)
*Actb*	NM_007393.3	F: AACGGCTCCGGCATGTGCAAAG, R: ATCACACCCTGGTGCCTAGGGCG	200 nM	55–61°C	–	–
*Avp*	NM_009732.1	F: CTGCTGGCCTTCTCCTCCGCC, R: CGGGCCGCAGGGGAGACAC	200 nM	58°C	7.59	2.30
*Ccl19*	NM_011888.2	F: ACCTCCAGACCAGCCCTGGGT, R: TGGTGCTGTTGCCTTTGTTCTTGGC	200 nM	61°C	1.36	1.72
*Hcrt*	NM_010410.2	F: TGGGTATTTGGACCACTGCACTGA, R: CAGGGAACCTTTGTAGAAGGAAAGTTC	200 nM	55°C	6.12	2.05
*Oxt*	NM_011025.3	F: TCACCTACAGCGGATCTCAGACTGA, R: CCCAGGGGGCAGTTCTGGATGTA	200 nM	55°C	18.9	3.37
*Trh*	NM_009426.2	F: CCAGGAGGAAGGTGCTGTGAC, R: GTGATCCAGGAATCTAAGGCAGC	200 nM	55°C	8.46	2.10
*Zbtb16*	NM_001033324.2	F: GTCCGGTCCGGTCCCCTC, R: GGGCTCAGGCATGGGGCTCT	200 nM	58°C	3.75	1.61

F: forward primer, R: reverse primer. Values represent mean of the adjusted fold difference *(aFD)* statistic between ‘peak’ and ‘trough’ samples. Positive *aFD* values indicate higher expression in the hypertensive group samples collected in the active period, and negative *aFD* values indicate higher expression in the hypertensive group samples collected in the inactive period.

The *a*FD value was used to compare ‘trough’ and ‘peak’ BP hypertensive samples. Normotensive samples collected at the same times of 24 h BP variation as for the hypertensive mice were used as controls. The statistical package SPSS for Windows, Release 17.0, was used to compare normotensive and hypertensive groups collected at both ‘peak’ and ‘trough’ BP by one-way analysis of variance (ANOVA), followed by correction for multiple testing using the Bonferroni post-hoc test, to determine significance of differences between the groups. Significance was set at *P*<0.05.

## Results

**Figure 2:**
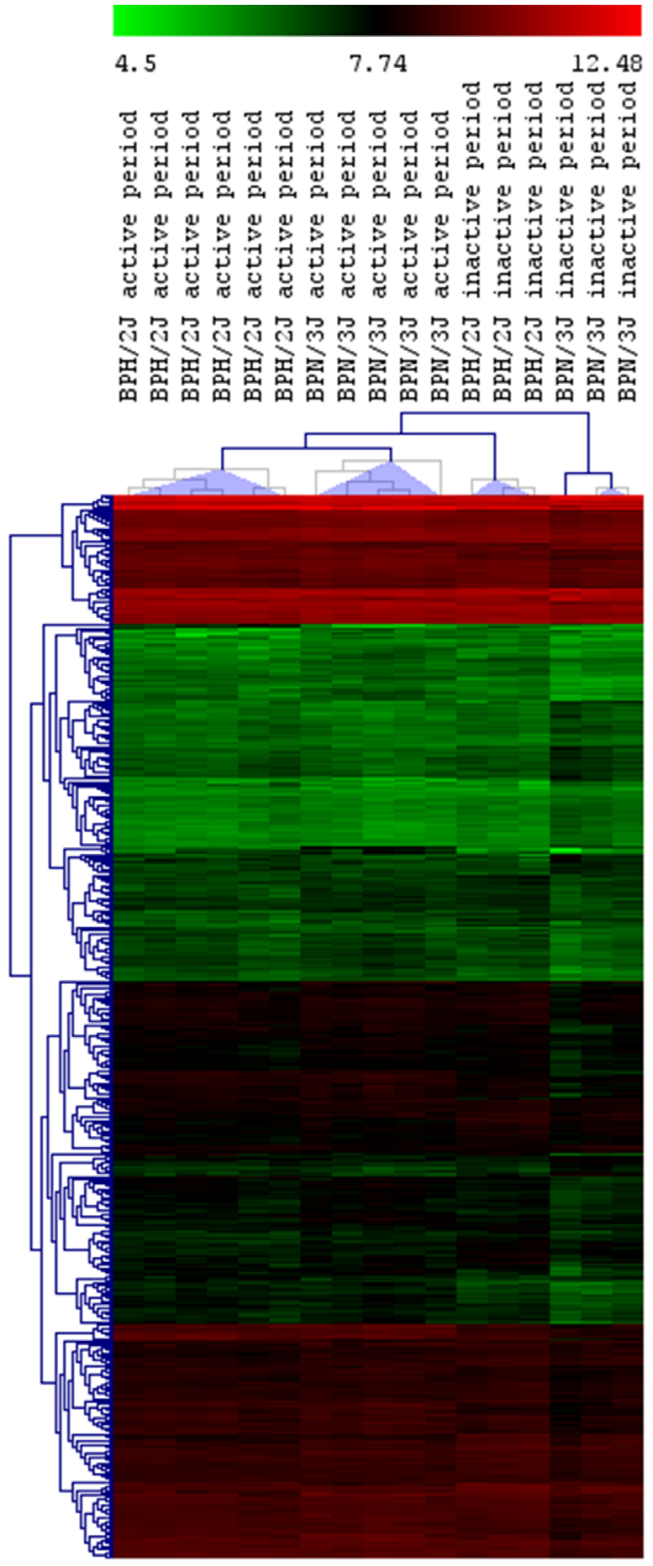
Hierarchical clustering comparing hypothalamic gene expression in active and inactive Schlager hypertensive and normotensive strains. Hierarchical clustering using Euclidean distance comparing the gene expression in the hypothalamus of ‘peak’ (left column, active period) versus ‘trough’ (right column, inactive period) hypertensive BPH/2J mice and age-matched normotensive BPN/3J samples. Distinctive patterns can be observed. Clusters of genes of similar biological relevance are indicated. Red depicts genes upregulated and green those downregulated.

**Figure 3:**
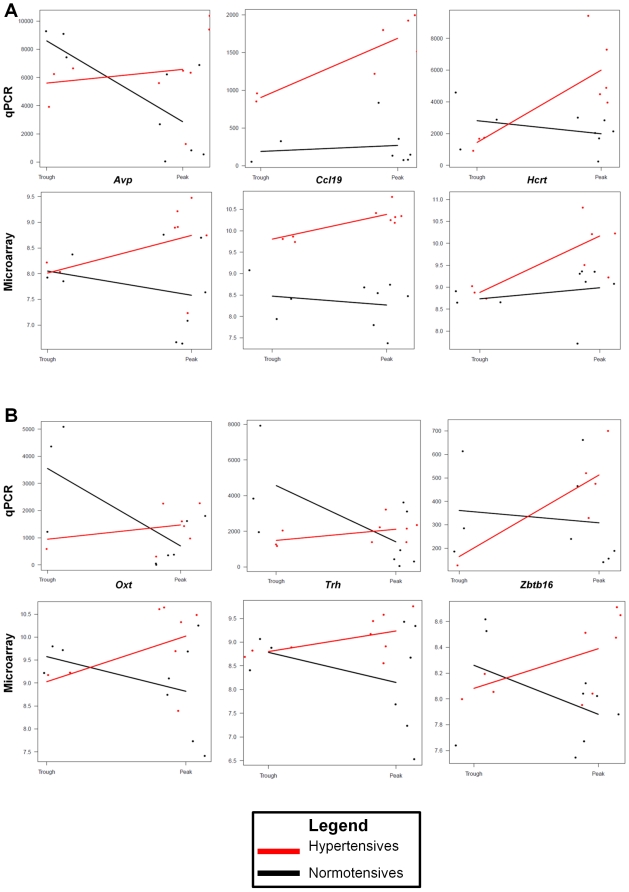
Validation of the *aFD* values using qPCR, showing results for the genes (A) *Avp*, *Ccl19*, *Hcrt*, and (B) *Oxt*, *Trh* and *Zbtb16*. This analysis took into account qPCR confirmation and the interaction between qPCR (top plots) and microarray results (bottom plots) with blood pressure (shown at the left of each plot is ‘trough’, and at the right is ‘peak’).

Hierarchical clustering showed that hypertensive and normotensive samples collected at ‘peak’ or ‘trough’ BP have distinctive patterns of gene expression (Figure 2). Using an *aFD* value ≥1.5, 212 well-annotated genes were identified between ‘trough’ and ‘peak’ BP samples of BPH/2J hypertensive mice. Table 1 summarizes the data for selected genes that we validated by qPCR (for complete information please see Table S1). *aFD* values from qPCR and microarray experiments are shown in Table 1 and Figure 3.

**Figure 4:**
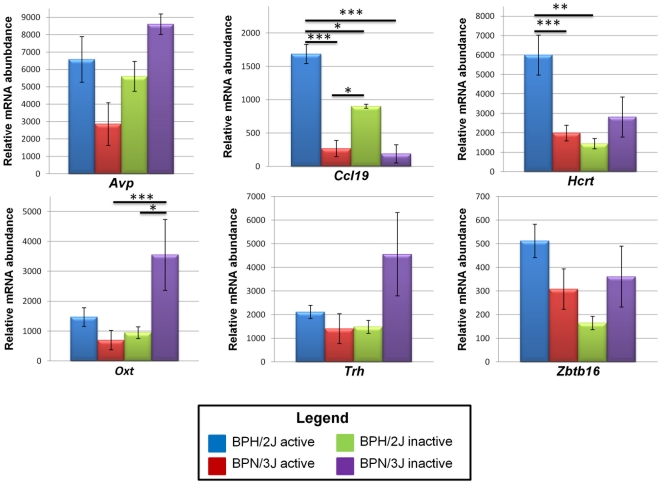
qPCR results for genes differentially expressed in the hypothalamus at ‘peak’ and ‘trough’ time-points in the Schlager hypertensive and normotensive strains. Shown is relative mRNA abundance for the genes *Avp*, *Ccl19*, *Hcrt*, *Oxt*, *Trh* and *Zbtb16*. Vertical bars show standard error of the mean; *P<0.05, **P<0.01, ***P<0.001.

ANOVA (Figure 4) showed that the hypertensive strain has an impaired response during the inactive period for the gene oxytocin (*Oxt*). Moreover the expression of the genes chemokine (C-C motif) ligand 19 (*Ccl19*) and hypocretin (*Hcrt*) was consistently higher during the active period in the BPH/2J than in the same strain during the inactive period or than the BPN/3J strain during the same period.

At ‘peak’ BP, GO analysis showed an enrichment of terms such as neuropeptide signaling pathway, defense response, chemokine and cytokine activity, immune system development (which could indicate an increase in inflammation), mitochondrial proton-transporting ATP synthase complex, and many terms related to structural constituent of ribosome, among others (see Table S2). At ‘peak’ BP in the BPH/2J hypertensive mice, the GST indicated an over-representation of G-protein coupled receptor protein signaling pathway, transcription factor and regulator activity, and cytokine activity, and a down-representation of chromatin modification, assembly or disassembly and helicase activity, amongst others (see Table S3).

**Figure 5:**
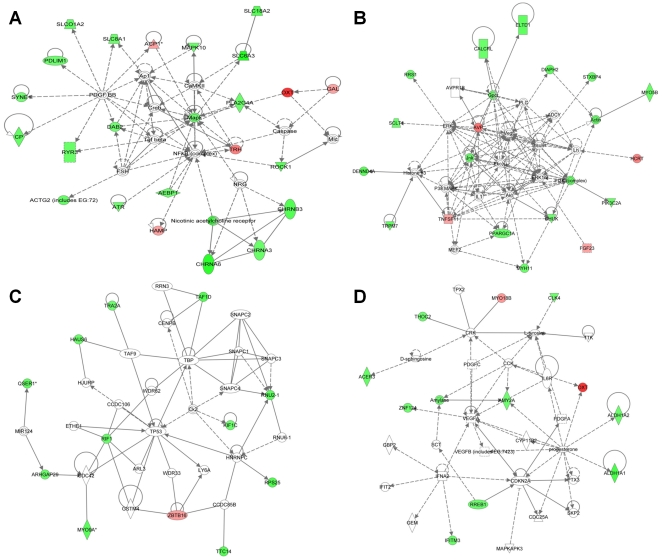
Top four molecular networks from the differentially expressed genes at ‘peak’ BP in the Schlager hypertensive mice, showing an enrichment of genes for (A) cardiovascular disease and molecular transport, (B) metabolic disease, (C) cell morphology, cellular assembly and organization, and (D) lipid metabolism, small molecule biochemistry and amino acid metabolism. The networks were constructed using the Ingenuity Pathway Analysis (IPA, Ingenuity® Systems, www.ingenuity.com) application. Genes over-expressed in our gene list are represented by green and genes under-expressed by red.

*In silico* molecular networks among the genes identified are shown in Figure 5. The most significant network highlighted cardiovascular disease and molecular transport (Figure 5A), consistent with a genetic component influencing circadian BP variation and thereby cardiovascular disease in this model.

## Discussion

This study is, to our knowledge, the first to evaluate genome-wide gene expression signatures in the hypothalamus during circadian variation of BP in the Schlager hypertensive mouse, a model that exhibits a similar morning BP surge as seen in human essential hypertension. The main findings suggest an involvement of arginine vasopressin (*Avp*), *Oxt* and thyrotropin releasing hormone (*Trh*), which are known genes in the regulation of BP. Our study proposes new candidates genes for the arousal-associated exaggerated circadian changes in BP in the BPH/2J mouse, such as *Ccl19*, *Hcrt*, and zinc finger and BTB domain containing 16 (*Zbtb16*). Consistent with the relevance of the BPH/2J strain to the morning BP surge in human hypertension, the present study has identified the genes for aldehyde dehydrogenase family 1 subfamily A2 (*Aldh1a2*) and solute carrier family 8 (sodium/calcium exchanger) member 1 (*Slc8a1*), which are two genes identified in genome-wide association studies of hypertension [22], [23]. All of these findings together, suggest indirect dysregulation of the angiotensinergic system and inflammation, amongst others, as influencing exaggerated circadian changes in BP in the Schlager hypertensive mouse.

Genes for catecholamine biosynthesis were not amongst those we identified. Nor were genes of the angiotensinergic system itself. On the other hand, the gene for *Zbtb16* (*aFD* = 1.6, also known as *Plzf*) can interact with and regulate components of the renin-angiotensin system. Following treatment with angiotensin II (Ang II), Zbtb16 binds to Ang II type 2 receptor (AGTR2) located in the plasma membrane, and then both internalize together [24]. Similarly, internalization is observed when prorenin/renin binds to the (pro)renin receptor, Atp6ba2 [25], [26]. Such binding activates the renin-angiotensin cascade and causes the translocation of Zbtb16 to the nucleus, where Zbtb16 represses transcription of *Atp6ap2* [25], [26]. This is consistent with an increase in angiotensinergic activity in the brain, resulting in increased Ang II formation [27] accompanied by elevation in binding of prorenin/renin to Atp6ap2. Therefore the overexpression of Zbtb16 observed here during the active phase might indicate that this gene is involved in a servo-regulatory mechanism that, by inhibiting component(s) of the renin-angiotensin system, could be attempting to bring BP back to normal levels.

Arginine vasopressin (AVP, encoded by the gene *Avp*, and whose mRNA showed an *aFD* of 2.3) and oxytocin (OXT; gene *Oxt*; *aFD* = 3.4) are evolutionarily-related hormones, AVP being a well-known regulator of body water balance and thereby BP [28]. AVP synthesis in the hypothalamus is increased by many stimuli, including hyperosmolality [29], Ang II or a decrease in BP [28], [30], although it remains to be seen whether the BP reduction during the ‘trough’ of circadian BP in the Schlager hypertensive mouse would be sufficient to contribute to the rise in *Avp* expression. Microinjection of oxytocin into the rostroventrolateral medulla increases BP [31], and the deletion of the gene *Avp* leads to hypotension [32]. Both AVP and OXT are co-localized with Atp6ap2 in the hypothalamus [33], consistent with the possible involvement of a brain angiotensinergic system in circadian BP regulation.

In support of our findings, overexpression of *Trh* (*aFD* = 2.1) leads to hypertension in normal rats [34]. In contrast, knocking down this gene reduced BP in obesity-induced hypertensive rats [35]. The effect of TRH on BP seems to be mediated by the effects on sympathetic nerve activity [36].

We have demonstrated recently that *Ccl19* (*aFD* = 1.7) and *Hcrt* (*aFD* = 2.1) are differentially expressed in BPH/2J mice in early and established phases of hypertension [37]. In the present study we have now shown that these genes also contribute to the exaggerated circadian BP differences in this mouse strain. The overexpression of *Ccl19* might increase inflammatory response by attracting lymphocytes and dendritic cells [38]. The increase in inflammation at ‘peak’ BP in the BPH/2J mouse is supported by our GO and GST analyses, and the higher levels of inflammatory markers that have been reported in hypertensive patients during the morning BP surge [39].

Besides having a possible role in the onset and maintenance of BP in hypertensive Schlager mice, *Hcrt* also seems to contribute to the heightened stress response in this strain [37] and now in circadian BP. Intracerebroventricular (i.c.v.) administration of hypocretin, by acting on PVN neurons, increases mean arterial pressure, heart rate and renal sympathetic nerve activity [40], [41]. *Hcrt* knockout experiments showed that endogenous hypocretin participates in BP maintenance [42], apparently by increasing sympathetic outflow and consequent induction of the sympatho-adrenomedullary system [42], [43].

None of the 212 genes we identified here were clock genes, highlighting the success of our statistical analysis in the elimination of clock genes. Moreover our data analysis shows the potency of the *aFD* statistics in microarray analysis, the latter being validated by qPCR. In the case of *Avp*, *Oxt* and *Trh*, the *aFD* value measured by qPCR was considerably larger than that generated by the microarray analysis. Such findings are not unique [44] and most likely represent differences between solid-state and solution hybridization, coupled with use of the RMA algorithm, which provides greater specificity and sensitivity, but blunts the magnitude of the fold change [14].

The present study was facilitated by the large magnitude of the circadian changes in BP characteristic of the genetic model of hypertension studied. Other commonly used genetic models of hypertension, such as the spontaneously hypertensive rat, would be much less suitable because they show much smaller circadian differences in BP [45]. Although the replication of our findings in humans would be desirable, biopsy of hypothalamic tissue from human subjects, especially at specific times of the diurnal cycle, would present a challenge. Animal experiments *in vivo* will, moreover, be necessary to discern whether the changes in expression of these genes have a role in the circadian variation in BP in the Schlager hypertensive mouse or if they merely reflect secondary or coincidental phenomena that are not causally influencing BP. Such investigations were beyond the scope of the present study.

Although we sought to prevent clock genes from showing up in our analysis, and no known clock genes were amongst the genes we identified, we cannot absolutely rule out the possibility that some of the genes found were ones not previously recognized as having clock functions, and thus could nevertheless also be contributing to the strain differences in circadian BP patterns between the hypertensive and normotensive mice.

In conclusion, the present study has identified hypothalamic gene signatures of exaggerated circadian BP changes in hypertension in the Schlager BPH/2J mouse, which displays a morning BP surge similar to that seen in human essential hypertension. The 212 differentially expressed genes identified included *Aldh1a2*, *Avp*, *Ccl19*, *Hcrt*, *Oxt*, *Slc8a1*, *Trh* and *Zbtb16*. The integration of pathways involved in the neural and endocrine communication of the hypothalamus with other tissues is highly complex, and much remains to be elucidated. The particular genes revealed here were, moreover, supported by a combination of validation by qPCR, biological meaning and the use of robust statistical analyses with stringent adjustments. Our findings should help guide further research aimed at elucidation of the mechanisms involved in the cause of circadian variation in BP in the Schlager hypertensive mouse and, ultimately, in the morning BP surge in human essential hypertension.

## Supporting Information

Table S1 Genes that differed between ‘peak’ and ‘trough’ BP in BPH/2J Schlager mice after correction by matched awake/asleep controls using an adjusted fold difference (*aFD*) value of ≥1.5.
(DOC)

Table S2 Gene ontology analysis of the gene list for hypertension in the hypothalamus of the Schlager BPH/2J mouse.
(DOC)

Table S3 Gene set tests, based on gene ontology, of the gene list for hypertension in the hypothalamus of the Schlager BPH/2J mouse.
(DOC)
