# Supplementary material for: Genes Influencing Circadian Differences in Blood Pressure in Hypertensive Mice
Source: PLoS One. 2011 Apr 26;6(4):e19203. doi: 10.1371/journal.pone.0019203 (PMC3082552; doi:10.1371/journal.pone.0019203)
Supplement: Table S1 — Genes that differed between ‘peak’ and ‘trough’ BP in BPH/2J Schlager mice after correction by matched awake/asleep controls using an adjusted fold difference (aFD) value of ≥1.5. (DOC) [file pone.0019203.s001.doc]

**Table S1.** Genes that differed between ‘peak’ and ‘trough’ BP in BPH/2J Schlager mice after correction by matched awake/asleep controls using an adjusted fold difference (*aFD*) value of ≥1.5.

| **Probeset ID** | **Entrez ID** | **Official gene symbol** | **Official gene name** | ***aFD* value** |
| --- | --- | --- | --- | --- |
| 10423358 | 211147 | *40603* | membrane-associated ring finger (C3HC4) 11 | –1.55 |
| 10490273 | 100043387 | *100043387* | predicted gene, 100043387 | –1.59 |
| 10599951 | 68618 | *1110012L19Rik* | RIKEN cDNA 1110012L19 gene | –1.56 |
| 10518335 | 70005 | *1700029I01Rik* | RIKEN cDNA 1700029I01 gene | –1.75 |
| 10461921 | 67383 | *2410127L17Rik* | RIKEN cDNA 2410127L17 gene | 1.51 |
| 10490302 | 353208 | *2810021G02Rik* | RIKEN cDNA 2810021G02 gene | –1.60 |
| 10350592 | 73176 | *3110040M04Rik* | RIKEN cDNA 3110040M04 gene | 1.54 |
| 10598192 | 74851 | *4930408F14Rik* | RIKEN cDNA 4930408F14 gene | 1.61 |
| 10532305 | 100041734 | *4930522L14Rik* | RIKEN cDNA 4930522L14 gene | –1.61 |
| 10491601 | 229227 | *4932438A13Rik* | RIKEN cDNA 4932438A13 gene | –1.51 |
| 10460123 | 319609 | *9330132A10Rik* | RIKEN cDNA 9330132A10 gene | –1.62 |
| 10527982 | 207686 | *A330021E22Rik* | RIKEN cDNA A330021E22 gene | –1.56 |
| 10536635 | 214642 | *A430107O13Rik* | RIKEN cDNA A430107O13 gene | –1.53 |
| 10399299 | 207921 | *A830093I24Rik* | RIKEN cDNA A830093I24 gene | –1.76 |
| 10543785 | 232685 | *AB041803* | cDNA sequence AB041803 | –1.58 |
| 10392522 | 217258 | *Abca8a* | ATP-binding cassette, sub-family A (ABC1), member 8a | –1.67 |
| 10565712 | 66190 | *Acer3* | alkaline ceramidase 3 | –1.62 |
| 10355401 | 11431 | *Acp1* | acid phosphatase 1, soluble | 1.58 |
| 10467124 | 11475 | *Acta2* | actin, α2, smooth muscle, aorta | –1.60 |
| 10374083 | 11568 | *Aebp1* | AE binding protein 1 | –1.53 |
| 10398362 | 100303646 | *AF357355* | snoRNA AF357355 | –1.68 |
| 10461979 | 11668 | *Aldh1a1* | aldehyde dehydrogenase family 1, subfamily A1 | –1.89 |
| 10586865 | 19378 | *Aldh1a2* | aldehyde dehydrogenase family 1, subfamily A2 | –1.54 |
| 10501555 | 11722 | *Amy1* | amylase 1, salivary | –1.50 |
| 10427997 | 67434 | *Ankrd33b* | ankyrin repeat domain 33B | 1.79 |
| 10495685 | 214137 | *Arhgap29* | Rho GTPase activating protein 29 | –1.58 |
| 10451851 | 71703 | *Armcx3* | armadillo repeat containing, X-linked 3 | –1.83 |
| 10388254 | 11484 | *Aspa* | aspartoacylase | –1.56 |
| 10394366 | 320817 | *Atad2b* | ATPase family, AAA domain containing 2B | –1.53 |
| 10604799 | 320940 | *Atp11c* | ATPase, class VI, type 11C | –1.57 |
| 10592983 | 27425 | *Atp5l* | ATP synthase, H^+^ transporting, mitochondrial F0 complex, subunit g | –1.53 |
| 10587892 | 245000 | *Atr* | ataxia telangiectasia and Rad3 related | –1.57 |
| 10487685 | 11998 | *Avp* | arginine vasopressin | 2.30 |
| 10516079 | 100038759 | *C130018J17Rik* | RIKEN cDNA C130018J17 gene | 1.59 |
| 10368997 | 320644 | *C130030K03Rik* | RIKEN cDNA C130030K03 gene | –1.60 |
| 10484371 | 54598 | *Calcrl* | calcitonin receptor-like | –1.52 |
| 10578557 | 408022 | *Ccdc111* | coiled-coil domain containing 111 | –1.55 |
| 10469066 | 74186 | *Ccdc3* | coiled-coil domain containing 3 | –1.50 |
| 10512322 | 24047 | *Ccl19* | chemokine (C-C motif) ligand 19 | 1.72 |
| 10349442 | 72949 | *Ccnt2* | cyclin T2 | –1.57 |
| 10346960 | 227210 | *Ccnyl1* | cyclin Y-like 1 | –1.76 |
| 10380859 | 69131 | *Cdk12* | cyclin-dependent kinase 12 | –1.75 |
| 10503198 | 320790 | *Chd7* | chromodomain helicase DNA binding protein 7 | –1.83 |
| 10593756 | 110834 | *Chrna3* | cholinergic receptor, nicotinic, α polypeptide 3 | –1.54 |
| 10577980 | 11440 | *Chrna6* | cholinergic receptor, nicotinic, α polypeptide 6 | –2.18 |
| 10571170 | 108043 | *Chrnb3* | cholinergic receptor, nicotinic, β polypeptide 3 | –1.78 |
| 10467921 | 12675 | *Chuk* | conserved helix-loop-helix ubiquitous kinase | –1.51 |
| 10375820 | 12750 | *Clk4* | CDC like kinase 4 | –1.52 |
| 10406852 | 71994 | *Cnn3* | calponin 3, acidic | –1.55 |
| 10348963 | 241175 | *Cntnap5b* | contactin associated protein-like 5B | –1.60 |
| 10490989 | 12870 | *Cp* | ceruloplasmin | –1.63 |
| 10428522 | 239420 | *Csmd3* | CUB and Sushi multiple domains 3 | –1.71 |
| 10344813 | 211660 | *Cspp1* | centrosome and spindle pole associated protein 1 | –1.76 |
| 10485580 | 228410 | *Cstf3* | cleavage stimulation factor, 3' pre-RNA, subunit 3 | –1.63 |
| 10417319 | 320333 | *D830030K20Rik* | RIKEN cDNA D830030K20 gene | 1.70 |
| 10422728 | 13132 | *Dab2* | disabled homolog 2 (*Drosophila*) | –1.50 |
| 10570663 | 13236 | *Defa25* | defensin, α, 25 | 1.92 |
| 10570693 | 13239 | *Defa5* | defensin, α, 5 | 1.82 |
| 10586248 | 102442 | *Dennd4a* | DENN/MADD domain containing 4A | –1.74 |
| 10601616 | 54004 | *Diap2* | diaphanous homolog 2 (*Drosophila*) | –1.69 |
| 10354563 | 227058 | *Dnahc7b* | dynein, axonemal, heavy chain 7B | –1.52 |
| 10596257 | 235567 | *Dnajc13* | DnaJ (Hsp40) homolog, subfamily C, member 13 | –1.53 |
| 10591614 | 319899 | *Dock6* | dedicator of cytokinesis 6 | –1.75 |
| 10407072 | 74559 | *Elovl7* | ELOVL family member 7, elongation of long chain fatty acids (yeast) | –1.77 |
| 10496872 | 170757 | *Eltd1* | EGF, latrophilin seven transmembrane domain containing 1 | –1.67 |
| 10496359 | 59308 | *Emcn* | endomucin | –1.51 |
| 10353549 | 68187 | *Fam135a* | family with sequence similarity 135, member A | –1.57 |
| 10360542 | 66359 | *Fam36a* | family with sequence similarity 36, member A | –1.96 |
| 10594800 | 235461 | *Fam63b* | family with sequence similarity 63, member B | –1.58 |
| 10591112 | 270120 | *Fat3* | FAT tumor suppressor homolog 3 (*Drosophila*) | –1.81 |
| 10542028 | 64654 | *Fgf23* | fibroblast growth factor 23 | 1.51 |
| 10349947 | 14264 | *Fmod* | fibromodulin | –1.56 |
| 10464471 | 14419 | *Gal* | galanin | 1.52 |
| 10511629 | 672214 | *Gm10136* | ribosomal protein L26 pseudogene | –1.95 |
| 10518346 | 433791 | *Gm13251* | predicted gene 13251 | –1.50 |
| 10408070 | 665596 | *Gm13646* | predicted gene 13646 | 1.62 |
| 10595630 | 100039707 | *Gm2382* | predicted gene 2382 | –1.56 |
| 10423803 | 382986 | *Gm5213* | ferritin light chain 1 pseudogene | 1.79 |
| 10412897 | 632287 | *Gm7084* | predicted gene 7084 | 1.52 |
| 10412537 | 666890 | *Gm8348* | predicted gene 8348 | –1.70 |
| 10403246 | 667375 | *Gm8598* | predicted gene 8598 | 1.74 |
| 10519667 | 667986 | *Gm8912* | predicted gene 8912 | 1.52 |
| 10394929 | 668662 | *Gm9292* | predicted gene 9292 | 1.62 |
| 10584252 | 671003 | *Gm9513* | predicted gene 9513 | 1.51 |
| 10391744 | 237943 | *Gpatch8* | G patch domain containing 8 | –1.75 |
| 10383485 | 209318 | *Gps1* | G protein pathway suppressor 1 | 1.66 |
| 10562166 | 66438 | *Hamp2* | hepcidin antimicrobial peptide 2 | 1.57 |
| 10514201 | 230376 | *Haus6* | HAUS augmin-like complex, subunit 6 | –1.59 |
| 10566254 | 15129 | *Hbb-b1* | hemoglobin, β adult major chain | –1.76 |
| 10391273 | 15171 | *Hcrt* | hypocretin | 2.05 |
| 10435271 | 77446 | *Heg1* | HEG homolog 1 (zebrafish) | –1.55 |
| 10494402 | 15077 | *Hist2h3c1* | histone cluster 2, H3c1 | –1.53 |
| 10497325 | 15289 | *Hmgb1* | high mobility group box 1 | –1.54 |
| 10547469 | 406236 | *Hsn2* | hereditary sensory neuropathy, type II | –1.63 |
| 10584576 | 15481 | *Hspa8* | heat shock protein 8 | –1.75 |
| 10569017 | 66141 | *Ifitm3* | interferon induced transmembrane protein 3 | –1.56 |
| 10403034 | 111507 | *Igh* | immunoglobulin heavy chain complex | 1.89 |
| 10504106 | 16157 | *Il11ra1* | interleukin 11 receptor, α chain 1 | 1.64 |
| 10598798 | 22289 | *Kdm6a* | 4lysine (K)-specific demethylase 6A | –1.53 |
| 10377982 | 16562 | *Kif1c* | kinesin family member 1C | –1.52 |
| 10493889 | 73719 | *Lce1c* | late cornified envelope 1C | 1.55 |
| 10607429 | 280487 | *LOC280487* | pol polyprotein | –2.22 |
| 10608442 | 380994 | *LOC380994* | similar to Sycp3 like Y-linked | 2.13 |
| 10502778 | 99633 | *Lphn2* | latrophilin 2 | –1.67 |
| 10531866 | 26414 | *Mapk10* | mitogen-activated protein kinase 10 | –1.54 |
| 10564539 | 244049 | *Mctp2* | multiple C2 domains, transmembrane 2 | –1.51 |
| 10503723 | 100019 | *Mdn1* | midasin homolog (yeast) | –1.54 |
| 10452860 | 76890 | *Memo1* | mediator of cell motility 1 | –1.52 |
| 10458757 | 21761 | *Morf4l1* | mortality factor 4 like 1 | 1.52 |
| 10457357 | 75739 | *Mpp7* | membrane protein, palmitoylated 7 (MAGUK p55 subfamily member 7) | –1.55 |
| 10437885 | 17880 | *Myh11* | myosin, heavy polypeptide 11, smooth muscle | –1.57 |
| 10532628 | 74376 | *Myo18b* | myosin XVIIIb | 1.71 |
| 10456653 | 17919 | *Myo5b* | myosin VB | –1.59 |
| 10585972 | 270163 | *Myo9a* | myosin IXa | –1.73 |
| 10586724 | 93697 | *Narg2* | NMDA receptor-regulated gene 2 | –1.51 |
| 10346722 | 269198 | *Nbeal1* | neurobeachin like 1 | –1.75 |
| 10399198 | 27057 | *Ncoa4* | nuclear receptor coactivator 4 | –1.63 |
| 10495878 | 64580 | *Ndst4* | N-deacetylase/N-sulfotransferase (heparin glucosaminyl) 4 | –1.54 |
| 10378427 | 258701 | *Olfr401* | olfactory receptor 401 | 1.53 |
| 10373610 | 258315 | *Olfr767* | olfactory receptor 767 | 1.75 |
| 10467529 | 226115 | *Opalin* | oligodendrocytic myelin paranodal and inner loop protein | –1.60 |
| 10476192 | 18429 | *Oxt* | oxytocin | 3.37 |
| 10386548 | 230721 | *Pabpc4* | poly(A) binding protein, cytoplasmic 4 | 1.55 |
| 10455088 | 93882 | *Pcdhb11* | protocadherin β 11 | –1.59 |
| 10455108 | 93887 | *Pcdhb16* | protocadherin β 16 | –1.58 |
| 10455112 | 93888 | *Pcdhb17* | protocadherin β 17 | –1.55 |
| 10455118 | 93889 | *Pcdhb18* | protocadherin β 18 | –1.61 |
| 10455123 | 93890 | *Pcdhb19* | protocadherin β 19 | –1.76 |
| 10455135 | 93892 | *Pcdhb21* | protocadherin β 21 | –1.68 |
| 10467420 | 54132 | *Pdlim1* | PDZ and LIM domain 1 (elfin) | –1.51 |
| 10534085 | 18682 | *Phkg1* | phosphorylase kinase γ1 | –1.54 |
| 10439710 | 208177 | *Phldb2* | pleckstrin homology-like domain, family B, member 2 | –1.55 |
| 10567173 | 18704 | *Pik3c2a* | phosphatidylinositol 3-kinase, C2 domain containing, α polypeptide | –1.59 |
| 10559454 | 18724 | *Pira11* | paired-Ig-like receptor A11 | 1.64 |
| 10409551 | 18740 | *Pitx1* | paired-like homeodomain transcription factor 1 | 1.51 |
| 10358434 | 18783 | *Pla2g4a* | phospholipase A2, group IVA (cytosolic, calcium-dependent) | –1.54 |
| 10529977 | 19017 | *Ppargc1a* | peroxisome proliferative activated receptor, γ, coactivator 1 α | –1.56 |
| 10416266 | 19057 | *Ppp3cc* | protein phosphatase 3, catalytic subunit, γ isoform | 1.54 |
| 10396008 | 328110 | *Prpf39* | PRP39 pre-mRNA processing factor 39 homolog (yeast) | –1.54 |
| 10607484 | 211612 | *Ptchd1* | patched domain containing 1 | –1.52 |
| 10485622 | 99003 | *Qser1* | glutamine and serine rich 1 | –1.69 |
| 10488322 | 241694 | *Ralgapa2* | Ral GTPase activating protein, α subunit 2 (catalytic) | –1.56 |
| 10500683 | 57785 | *Rangrf* | RAN guanine nucleotide release factor | 1.66 |
| 10422194 | 74213 | *Rbm26* | RNA binding motif protein 26 | –1.55 |
| 10520388 | 381626 | *Rbm33* | RNA binding motif protein 33 | –1.72 |
| 10398360 | 75745 | *Rian* | RNA imprinted and accumulated in nucleus | –1.91 |
| 10472058 | 51869 | *Rif1* | Rap1 interacting factor 1 homolog (yeast) | –1.57 |
| 10440564 | 78913 | *Rnf160* | ring finger protein 160 | –1.60 |
| 10501567 | 67225 | *Rnpc3* | RNA-binding region (RNP1, RRM) containing 3 | –1.66 |
| 10500356 | 19844 | *Rnu1b1* | U1b1 small nuclear RNA | 1.58 |
| 10603736 | 19848 | *Rnu2* | U2 small nuclear RNA | –1.70 |
| 10544523 | 19872 | *Rny1* | RNA, Y1 small cytoplasmic, Ro-associated | –1.91 |
| 10457429 | 19877 | *Rock1* | Rho-associated coiled-coil containing protein kinase 1 | –1.70 |
| 10443492 | 19933 | *Rpl21* | ribosomal protein L21 | –1.59 |
| 10528474 | 68028 | *Rpl22l1* | ribosomal protein L22 like 1 | –1.51 |
| 10518356 | 19944 | *Rpl29* | ribosomal protein L29 | –1.82 |
| 10457731 | 66489 | *Rpl35* | ribosomal protein L35 | –1.68 |
| 10452384 | 27176 | *Rpl7a* | ribosomal protein L7A | 1.66 |
| 10491730 | 66475 | *Rps23* | ribosomal protein S23 | –2.15 |
| 10398451 | 75617 | *Rps25* | ribosomal protein S25 | –1.83 |
| 10516479 | 54127 | *Rps28* | ribosomal protein S28 | –1.58 |
| 10404612 | 68750 | *Rreb1* | ras responsive element binding protein 1 | –1.54 |
| 10344723 | 59014 | *Rrs1* | RRS1 ribosome biogenesis regulator homolog (*S. cerevisiae*) | –1.54 |
| 10485840 | 20192 | *Ryr3* | ryanodine receptor 3 | –1.55 |
| 10497971 | 67161 | *Sclt1* | sodium channel and clathrin linker 1 | –1.56 |
| 10411454 | 66212 | *Sec61b* | Sec61 β subunit | 2.01 |
| 10494369 | 107701 | *Sf3b4* | splicing factor 3b, subunit 4 | –1.51 |
| 10443459 | 20383 | *Sfrs3* | splicing factor, arginine/serine-rich 3 (SRp20) | –1.54 |
| 10387100 | 380702 | *Shisa6* | shisa homolog 6 (*Xenopus laevis*) | –1.60 |
| 10522388 | 231290 | *Slc10a4* | solute carrier family 10 (sodium/bile acid cotransporter family), member 4 | –1.55 |
| 10464370 | 214084 | *Slc18a2* | solute carrier family 18 (vesicular monoamine), member 2 | –1.78 |
| 10412773 | 218756 | *Slc4a7* | solute carrier family 4, sodium bicarbonate cotransporter, member 7 | –1.55 |
| 10597960 | 102680 | *Slc6a20a* | solute carrier family 6 (neurotransmitter transporter), member 20A | –1.64 |
| 10406050 | 13162 | *Slc6a3* | solute carrier family 6 (neurotransmitter transporter, dopamine), member 3 | –2.31 |
| 10453231 | 20541 | *Slc8a1* | solute carrier family 8 (sodium/calcium exchanger), member 1 | –1.56 |
| 10548996 | 28250 | *Slco1a4* | solute carrier organic anion transporter family, member 1a4 | –1.60 |
| 10466682 | 226026 | *Smc5* | structural maintenance of chromosomes 5 | –1.60 |
| 10461158 | 83673 | *Snhg1* | small nucleolar RNA host gene (non-protein coding) 1 | –1.70 |
| 10564017 | 493919 | *Snord115* | Small nucleolar RNA, C/D Box 115 cluster | –1.91 |
| 10564165 | 64243 | *Snord116* | small nucleolar RNA, C/D box 116 cluster | –1.92 |
| 10586168 | 100217453 | *Snord16a* | small nucleolar RNA, C/D box 16A | –1.95 |
| 10563114 | 27209 | *Snord32a* | small nucleolar RNA, C/D box 32A | –1.63 |
| 10563112 | 27208 | *Snord33* | small nucleolar RNA, C/D box 33 | –1.67 |
| 10608382 | 385550 | *Srsy* | serine-rich, secreted, Y-linked | –1.55 |
| 10389797 | 20913 | *Stxbp4* | syntaxin binding protein 4 | –1.58 |
| 10599925 | 56291 | *Styx* | serine/threonine/tyrosine interaction protein | –1.52 |
| 10551531 | 68416 | *Sycn* | syncollin | 1.53 |
| 10361509 | 64009 | *Syne1* | synaptic nuclear envelope 1 | –1.51 |
| 10583316 | 75316 | *Taf1d* | TATA box binding protein (Tbp)-associated factor, RNA polymerase I, D | –1.83 |
| 10414960 | 21473 | *Tcra* | T-cell receptor α chain | 1.57 |
| 10360580 | 15278 | *Tfb2m* | transcription factor B2, mitochondrial | –1.55 |
| 10453276 | 240174 | *Thada* | thyroid adenoma associated | –1.52 |
| 10604248 | 331401 | *Thoc2* | THO complex 2 | –1.52 |
| 10525744 | 56334 | *Tmed2* | transmembrane emp24 domain trafficking protein 2 | –1.61 |
| 10421737 | 21943 | *Tnfsf11* | tumor necrosis factor (ligand) superfamily, member 11 | 1.52 |
| 10544638 | 101214 | *Tra2a* | transformer 2 α homolog (*Drosophila*) | –1.51 |
| 10546417 | 22044 | *Trh* | thyrotropin releasing hormone | 2.10 |
| 10498620 | 66949 | *Trim59* | tripartite motif-containing 59 | –2.16 |
| 10368511 | 73681 | *Trmt11* | tRNA methyltransferase 11 homolog (*S. cerevisiae*) | –1.61 |
| 10487277 | 58800 | *Trpm7* | transient receptor potential cation channel, subfamily M, member 7 | –1.66 |
| 10491438 | 67120 | *Ttc14* | tetratricopeptide repeat domain 14 | –1.79 |
| 10376864 | 22187 | *Ubb* | ubiquitin B | –1.89 |
| 10356423 | 227334 | *Usp40* | ubiquitin specific peptidase 40 | –1.54 |
| 10373692 | 625109 | *Vmn2r86* | vomeronasal 2, receptor 86 | –2.11 |
| 10571415 | 52348 | *Vps37a* | vacuolar protein sorting 37A (yeast) | –1.51 |
| 10587942 | 24127 | *Xrn1* | 5'-3' exoribonuclease 1 | –1.62 |
| 10593225 | 235320 | *Zbtb16* | zinc finger and BTB domain containing 16 | 1.61 |
| 10359377 | 240869 | *Zbtb37* | zinc finger and BTB domain containing 37 | –1.57 |
| 10604424 | 208968 | *Zfp280c* | zinc finger protein 280C | –1.61 |
| 10442238 | 22709 | *Zfp51* | zinc finger protein 51 | –1.63 |
| 10410650 | 235956 | *Zfp825* | zinc finger protein 825 | –1.53 |
| 10468881 | 414758 | *Zfp826* | zinc finger protein 826 | –1.59 |

*Values represent mean of the adjusted fold difference *(aFD)* statistic between ‘peak’ and ‘trough’ samples, based on an absolute *aFD* value exceeding ±1.50. Positive *aFD* values indicate higher expression in the hypertensive group collected in the active period and negative *aFD* values indicate higher expression in hypertensive group collected in the inactive period.
