# Supplementary material for: Genes Influencing Circadian Differences in Blood Pressure in Hypertensive Mice
Source: PLoS One. 2011 Apr 26;6(4):e19203. doi: 10.1371/journal.pone.0019203 (PMC3082552; doi:10.1371/journal.pone.0019203)
Supplement: Table S2 — Gene ontology analysis of the gene list for hypertension in the hypothalamus of the Schlager BPH/2J mouse. (DOC) [file pone.0019203.s002.doc]

**Table S2.** Gene ontology analysis of the gene list for hypertension in the hypothalamus of the Schlager BPH/2J mouse.

| **Ontology** | **Representation** | **GOCCID** | **Adjusted *P* value** | **Odds Ratio** | **Count** | **Size** | **Term** | **Genes** |
| --- | --- | --- | --- | --- | --- | --- | --- | --- |
| BP | Over | GO:0042742 | 0.18 | 26 | 3 | 32 | defense response to bacterium | *Defa25, Defa5, Hamp2* |
| BP | Over | GO:0007218 | 0.18 | 16 | 3 | 50 | neuropeptide signaling pathway | *Gal, Hcrt, Lphn2* |
| BP | Over | GO:0045639 | 0.18 | 32 | 2 | 17 | positive regulation of myeloid cell differentiation | *Hmgb1, Tnfsf11* |
| BP | Over | GO:0009617 | 0.18 | 12 | 3 | 65 | response to bacterium | *Defa25, Defa5, Hamp2* |
| BP | Over | GO:0035116 | 0.18 | 27 | 2 | 20 | embryonic hindlimb morphogenesis | *Pitx1, Zbtb16* |
| BP | Over | GO:0035137 | 0.18 | 21 | 2 | 25 | hindlimb morphogenesis | *Pitx1, Zbtb16* |
| BP | Over | GO:0051704 | 0.18 | 6.0 | 4 | 170 | multi-organism process | *Defa25, Defa5, Hamp2*  *Il11ra1* |
| BP | Over | GO:0051707 | 0.18 | 7.9 | 3 | 97 | response to other organism | *Defa25, Defa5, Hamp2* |
| BP | Over | GO:0006952 | 0.18 | 5.3 | 4 | 193 | defense response | *Ccl19, Defa25, Defa5, Hamp2* |
| BP | Over | GO:0007608 | 0.18 | 15 | 2 | 35 | sensory perception of smell | *Olfr401, Olfr767* |
| BP | Over | GO:0045637 | 0.18 | 14 | 2 | 36 | regulation of myeloid cell differentiation | *Hmgb1, Tnfsf11* |
| BP | Over | GO:0030097 | 0.18 | 4.9 | 4 | 209 | hemopoiesis | *Hmgb1, Il11ra1, Tcra, Tnfsf11* |
| BP | Over | GO:0007606 | 0.18 | 13 | 2 | 39 | sensory perception of chemical stimulus | *Olfr401, Olfr767* |
| BP | Over | GO:0001501 | 0.18 | 4.7 | 4 | 218 | skeletal system development | *Fgf23, Pitx1, Tnfsf11, Zbtb16* |
| BP | Over | GO:0008283 | 0.18 | 3.3 | 6 | 468 | cell proliferation | *Hmgb1, Il11ra1, Morf4l1, Rpl29, Tnfsf11, Zbtb16* |
| BP | Over | GO:0048534 | 0.18 | 4.5 | 4 | 224 | hemopoietic or lymphoid organ development | *Hmgb1, Il11ra1, Tcra, Tnfsf11* |
| BP | Over | GO:0002521 | 0.18 | 6.1 | 3 | 124 | leukocyte differentiation | *Il11ra1, Tcra, Tnfsf11* |
| BP | Over | GO:0043193 | 0.18 | 11 | 2 | 46 | positive regulation of gene-specific transcription | *Fgf23, Hmgb1* |
| BP | Over | GO:0007186 | 0.19 | 3.6 | 5 | 358 | G-protein coupled receptor protein signaling pathway | *Gal, Hcrt, Lphn2, Olfr401, Olfr767* |
| BP | Over | GO:0002520 | 0.19 | 4.3 | 4 | 236 | immune system development | *Hmgb1, Il11ra1, Tcra, Tnfsf11* |
| BP | Over | GO:0010740 | 0.19 | 10 | 2 | 50 | positive regulation of protein kinase cascade | *Fgf23, Tnfsf11* |
| BP | Over | GO:0002360 | 0.19 | 60 | 1 | 5 | T cell lineage commitment | *Tcra* |
| BP | Over | GO:0010676 | 0.19 | 60 | 1 | 5 | positive regulation of cellular carbohydrate metabolic process | *Hmgb1* |
| BP | Over | GO:0010907 | 0.19 | 60 | 1 | 5 | positive regulation of glucose metabolic process | *Hmgb1* |
| BP | Over | GO:0043470 | 0.19 | 60 | 1 | 5 | regulation of carbohydrate catabolic process | *Hmgb1* |
| BP | Over | GO:0043471 | 0.19 | 60 | 1 | 5 | regulation of cellular carbohydrate catabolic process | *Hmgb1* |
| BP | Over | GO:0045913 | 0.19 | 60 | 1 | 5 | positive regulation of carbohydrate metabolic process | *Hmgb1* |
| BP | Over | GO:0006935 | 0.19 | 9.5 | 2 | 53 | chemotaxis | *Ccl19, Hmgb1* |
| BP | Over | GO:0042330 | 0.19 | 9.5 | 2 | 53 | taxis | *Ccl19, Hmgb1* |
| BP | Over | GO:0009607 | 0.20 | 5.2 | 3 | 145 | response to biotic stimulus | *Defa25, Defa5, Hamp2* |
| BP | Over | GO:0001893 | 0.20 | 48 | 1 | 6 | maternal placenta development | *Il11ra1* |
| BP | Over | GO:0030816 | 0.20 | 48 | 1 | 6 | positive regulation of cAMP metabolic process | *Avp* |
| BP | Over | GO:0030819 | 0.20 | 48 | 1 | 6 | positive regulation of cAMP biosynthetic process | *Avp* |
| BP | Over | GO:0031960 | 0.20 | 48 | 1 | 6 | response to corticosteroid stimulus | *Hmgb1* |
| BP | Over | GO:0050926 | 0.20 | 48 | 1 | 6 | regulation of positive chemotaxis | *Hmgb1* |
| BP | Over | GO:0050927 | 0.20 | 48 | 1 | 6 | positive regulation of positive chemotaxis | *Hmgb1* |
| BP | Over | GO:0050930 | 0.20 | 48 | 1 | 6 | induction of positive chemotaxis | *Hmgb1* |
| BP | Over | GO:0051384 | 0.20 | 48 | 1 | 6 | response to glucocorticoid stimulus | *Hmgb1* |
| CC | Over | GO:0005576 | 0.003 | 4.4 | 12 | 769 | extracellular region | *Avp, Ccl19, Defa25, Defa5, Fgf23, Gal, Hamp2, Hmgb1, Oxt, Sftpa1, Tnfsf11, Trh* |
| CC | Over | GO:0005840 | 0.003 | 9.2 | 6 | 174 | ribosome | *Rpl21, Rpl29, Rpl7a, Rps23, Rps25, Rps28* |
| CC | Over | GO:0005844 | 0.02 | 68 | 2 | 9 | polysome | *Rpl29, Rpl7a* |
| CC | Over | GO:0005615 | 0.02 | 6.8 | 5 | 190 | extracellular space | *Ccl19, Defa25, Defa5, Hmgb1, Tnfsf11* |
| CC | Over | GO:0030529 | 0.02 | 4.6 | 7 | 394 | ribonucleoprotein complex | *Rpl21, Rpl29, Rpl7a, Rps23, Rps25, Rps28, Sf3b4* |
| CC | Over | GO:0044421 | 0.06 | 4.2 | 6 | 363 | extracellular region part | *Ccl19, Defa25, Defa5, Hmgb1, Sftpa1, Tnfsf11* |
| CC | Over | GO:0032991 | 0.17 | 2.1 | 14 | 1765 | macromolecular complex | *Atp5l, Gm13646, Gps1, Morf4l1, Myo18b, Rpl21, Rpl29, Rpl7a, Rps23, Rps25, Rps28, Sf3b4, Tcra, Zbtb16* |
| CC | Over | GO:0000276 | 0.19 | 59 | 1 | 5 | mitochondrial proton-transporting ATP synthase complex, coupling factor F(o) | *Atp5l* |
| CC | Over | GO:0005625 | 0.19 | 8.9 | 2 | 56 | soluble fraction | *Cycs, Hmgb1* |
| CC | Over | GO:0005753 | 0.19 | 47 | 1 | 6 | mitochondrial proton-transporting ATP synthase complex | *Atp5l* |
| CC | Down | GO:0016459 | 0.07 | 8.9 | 4 | 37 | myosin complex | *Myh11, Myo18b, Myo5b, Myo9a* |
| CC | Down | GO:0005892 | 0.07 | 49 | 2 | 5 | nicotinic acetylcholine-gated receptor-channel complex | *Chrna3, Chrna6* |
| CC | Down | GO:0005840 | 0.09 | 3.6 | 8 | 174 | ribosome | *Rpl21, Rpl22l1, Rpl29, Rpl35, Rpl7a, Rps23, Rps25, Rps28* |
| CC | Down | GO:0044449 | 0.14 | 6.2 | 4 | 51 | contractile fiber part | *Acta2, Myh11, Myo18b, Syne1* |
| CC | Down | GO:0005844 | 0.14 | 21 | 2 | 9 | polysome | *Rpl29, Rpl7a* |
| CC | Down | GO:0043292 | 0.15 | 5.5 | 4 | 57 | contractile fiber | *Acta2, Myh11, Myo18b, Syne1* |
| MF | Over | GO:0005179 | *P*<0.001 | 33 | 5 | 46 | hormone activity | *Avp, Gal, Hamp2, Oxt, Trh* |
| MF | Over | GO:0005102 | 0.001 | 7.8 | 9 | 347 | receptor binding | *Avp, Ccl19, Fgf23, Gal, Hamp2, Hmgb1, Oxt, Tnfsf11, Trh* |
| MF | Over | GO:0005184 | 0.001 | 71 | 3 | 14 | neuropeptide hormone activity | *Avp, Oxt, Trh* |
| MF | Over | GO:0005125 | 0.04 | 14 | 3 | 60 | cytokine activity | *Ccl19, Hmgb1, Tnfsf11* |
| MF | Over | GO:0003735 | 0.04 | 8.2 | 4 | 133 | structural constituent of ribosome | *Rpl21, Rpl29, Rps23, Rps28* |
| MF | Over | GO:0004984 | 0.04 | 24 | 2 | 23 | olfactory receptor activity | *Olfr401, Olfr767* |
| MF | Over | GO:0005126 | 0.10 | 11 | 2 | 47 | cytokine receptor binding | *Ccl19, Tnfsf11* |
| MF | Over | GO:0008187 | 0.12 | 63 | 1 | 5 | poly-pyrimidine tract binding | *Pabpc4* |
| MF | Over | GO:0008201 | 0.12 | 9.3 | 2 | 57 | heparin binding | *Hmgb1, Rpl29* |
| MF | Over | GO:0030246 | 0.12 | 5.2 | 3 | 151 | carbohydrate binding | *Hmgb1, Rpl29, Sftpa1* |
| MF | Over | GO:0005164 | 0.12 | 50 | 1 | 6 | tumor necrosis factor receptor binding | *Tnfsf11* |
| MF | Over | GO:0005198 | 0.12 | 3.8 | 4 | 281 | structural molecule activity | *Rpl21, Rpl29, Rps23, Rps28* |
| MF | Over | GO:0003993 | 0.12 | 42 | 1 | 7 | acid phosphatase activity | *Acp1* |
| MF | Over | GO:0008143 | 0.12 | 42 | 1 | 7 | poly(A) RNA binding | *Pabpc4* |
| MF | Over | GO:0008536 | 0.12 | 42 | 1 | 7 | Ran GTPase binding | *Rangrf* |
| MF | Over | GO:0070717 | 0.12 | 42 | 1 | 7 | poly-purine tract binding | *Pabpc4* |
| MF | Over | GO:0030295 | 0.15 | 31 | 1 | 9 | protein kinase activator activity | *Hmgb1* |
| MF | Over | GO:0032813 | 0.15 | 31 | 1 | 9 | tumor necrosis factor receptor superfamily binding | *Tnfsf11* |
| MF | Over | GO:0005539 | 0.15 | 6.9 | 2 | 76 | glycosaminoglycan binding | *Hmgb1, Rpl29* |
| MF | Over | GO:0008009 | 0.15 | 28 | 1 | 10 | chemokine activity | *Ccl19* |
| MF | Over | GO:0001871 | 0.15 | 6.4 | 2 | 82 | pattern binding | *Hmgb1, Rpl29* |
| MF | Over | GO:0030247 | 0.15 | 6.4 | 2 | 82 | polysaccharide binding | *Hmgb1, Rpl29* |
| MF | Over | GO:0042379 | 0.15 | 25 | 1 | 11 | chemokine receptor binding | *Ccl19* |
| MF | Over | GO:0005104 | 0.16 | 23 | 1 | 12 | fibroblast growth factor receptor binding | *Fgf23* |
| MF | Over | GO:0008553 | 0.16 | 23 | 1 | 12 | hydrogen-exporting ATPase activity, phosphorylative mechanism | *Atp5l* |
| MF | Over | GO:0003727 | 0.16 | 21 | 1 | 13 | single-stranded RNA binding | *Pabpc4* |
| MF | Over | GO:0019209 | 0.16 | 21 | 1 | 13 | kinase activator activity | *Hmgb1* |
| MF | Over | GO:0043022 | 0.16 | 21 | 1 | 13 | ribosome binding | *Sec61b* |
| MF | Over | GO:0016566 | 0.18 | 18 | 1 | 15 | specific transcriptional repressor activity | *Zbtb16* |
| MF | Down | GO:0004889 | 0.02 | 55 | 3 | 7 | nicotinic acetylcholine-activated cation-selective channel activity | *Chrna3, Chrna6, Chrnb3* |
| MF | Down | GO:0005516 | 0.13 | 5.0 | 6 | 94 | calmodulin binding | *Cnn3, Myh11, Myo5b, Phkg1, Ppp3cc, Slc8a1* |
| MF | Down | GO:0015370 | 0.16 | 9.9 | 3 | 25 | solute:sodium symporter activity | *Slc10a4, Slc6a20a, Slc6a3* |
| MF | Down | GO:0003774 | 0.16 | 4.7 | 5 | 83 | motor activity | *Kif1c, Myh11, Myo18b, Myo5b, Myo9a* |
| MF | Down | GO:0015075 | 0.16 | 2.2 | 13 | 450 | ion transmembrane transporter activity | *Atp11c, Atp5l, Chrna3, Chrna6, Chrnb3, Ryr3, Slc10a4, Slc4a7, Slc6a20a, Slc6a3, Slc8a1, Slco1a4, Trpm7* |
| MF | Down | GO:0005231 | 0.16 | 7.5 | 3 | 32 | excitatory extracellular ligand-gated ion channel activity | *Chrna3, Chrna6, Chrnb3* |
| MF | Down | GO:0003735 | 0.16 | 3.5 | 6 | 133 | structural constituent of ribosome | *Rpl21, Rpl22l1, Rpl29, Rpl35, Rps23, Rps28* |
| MF | Down | GO:0005328 | 0.16 | 14 | 2 | 12 | neurotransmitter:sodium symporter activity | *Slc6a20a, Slc6a3* |
| MF | Down | GO:0005326 | 0.16 | 12 | 2 | 14 | neurotransmitter transporter activity | *Slc6a20a, Slc6a3* |
| MF | Down | GO:0015294 | 0.16 | 5.7 | 3 | 41 | solute:cation symporter activity | *Slc10a4, Slc6a20a, Slc6a3* |
| MF | Down | GO:0015291 | 0.16 | 3.4 | 5 | 113 | secondary active transmembrane transporter activity | *Slc10a4, Slc4a7, Slc6a20a, Slc6a3, Slc8a1* |
| MF | Down | GO:0022891 | 0.16 | 2.0 | 13 | 506 | substrate-specific transmembrane transporter activity | *Atp11c, Atp5l, Chrna3, Chrna6, Chrnb3, Ryr3* |
| MF | Down | GO:0022804 | 0.16 | 2.5 | 7 | 208 | active transmembrane transporter activity | *Atp11c, Atp5l, Slc10a4, Slc4a7, Slc6a20a, Slc6a3* |
| MF | Down | GO:0005230 | 0.16 | 5.0 | 3 | 46 | extracellular ligand-gated ion channel activity | *Chrna3, Chrna6, Chrnb3* |
| MF | Down | GO:0015293 | 0.16 | 3.8 | 4 | 81 | symporter activity | *Slc10a4, Slc4a7, Slc6a20a, Slc6a3* |
| MF | Down | GO:0022892 | 0.19 | 1.8 | 14 | 588 | substrate-specific transporter activity | *Atp11c, Atp5l, Chrna3, Chrna6, Chrnb3, Hbb-b1, Ryr3, Slc10a4, Slc4a7, Slc6a20a, Slc6a3, Slc8a1, Slco1a4, Trpm7* |
| MF | Down | GO:0003779 | 0.20 | 2.4 | 7 | 224 | actin binding | *Cnn3, Diap2, Myh11, Myo5b, Myo9a, Syne1, Trpm7* |
| MF | Down | GO:0016620 | 0.20 | 7.2 | 2 | 22 | oxidoreductase activity, acting on the aldehyde or oxo group of donors, NAD or NADP as acceptor | *Aldh1a1, Aldh1a2* |
| MF | Down | GO:0004674 | 0.20 | 2.1 | 9 | 328 | protein serine/threonine kinase activity | *Atr, Cdk12, Chuk, Clk4, Mapk10, Phkg1, Prkdc, Rock1, Trpm7* |
| MF | Down | GO:0005509 | 0.20 | 1.8 | 13 | 552 | calcium ion binding | *Amy1, Eltd1, Fat3, Mctp2, Pcdhb11, Pcdhb16, Pcdhb17, Pcdhb18, Pcdhb19, Pcdhb21, Pla2g4a, Slc8a1, Trpm7* |
| KEGG | Over | 3010 | 0.001 | 15 | 6 | 85 | Ribosome | *Rpl21, Rpl29, Rpl7a, Rps23, Rps25, Rps28* |
| KEGG | Over | 4060 | 0.26 | 5.0 | 3 | 96 | Cytokine-cytokine receptor interaction | *Ccl19, Il11ra1, Tnfsf11* |
| KEGG | Over | 4640 | 0.26 | 9.5 | 2 | 37 | Hematopoietic cell lineage | *Igh, Il11ra1* |
| KEGG | Over | 4740 | 0.26 | 8.5 | 2 | 41 | Olfactory transduction | *Olfr401, Olfr767* |
| KEGG | Over | 5014 | 0.26 | 7.3 | 2 | 47 | Amyotrophic lateral sclerosis (ALS) | *Cycs, Ppp3cc* |
| KEGG | Over | 5310 | 0.26 | 32 | 1 | 6 | Asthma | *Igh* |
| KEGG | Over | 5416 | 0.26 | 6.9 | 2 | 50 | Viral myocarditis | *Cycs, Igh* |
| KEGG | Over | 4662 | 0.28 | 5.7 | 2 | 60 | B cell receptor signaling pathway | *Igh, Ppp3cc* |
| KEGG | Over | 5322 | 0.28 | 5.7 | 2 | 60 | Systemic lupus erythematosus | *Gm13646, Igh* |
| KEGG | Over | 740 | 0.28 | 16 | 1 | 11 | Riboflavin metabolism | *Acp1* |
| KEGG | Over | 4210 | 0.28 | 5.0 | 2 | 68 | Apoptosis | *Cycs, Ppp3cc* |
| KEGG | Over | 4650 | 0.28 | 4.8 | 2 | 70 | Natural killer cell mediated cytotoxicity | *Igh, Ppp3cc* |
| KEGG | Down | 3010 | *P*<0.001 | 8.2 | 8 | 85 | Ribosome | *Rpl21, Rpl22l1, Rpl29, Rpl35, Rpl7a, Rps23, Rps25, Rps28* |

*Ontology: BP, biological process; MF, molecular function; GOID: Gene Ontology identification. Shown is the Benjamini-Hochberg false discovery rate-adjusted *P* value; OR, odds ratio: p(Ontology W in the list)/p(Ontology W in the universe); ExpCount: expected count; Count: number of genes found that belong to ontology; Size: total number of genes in ontology; Term: ontology-associated term; Gene Symbol: official gene symbol.
