# Supplementary material for: Genes Influencing Circadian Differences in Blood Pressure in Hypertensive Mice
Source: PLoS One. 2011 Apr 26;6(4):e19203. doi: 10.1371/journal.pone.0019203 (PMC3082552; doi:10.1371/journal.pone.0019203)
Supplement: Table S3 — Gene set tests, based on gene ontology, of the gene list for hypertension in the hypothalamus of the Schlager BPH/2J mouse. (DOC) [file pone.0019203.s003.doc]

**Table S3.** Gene set tests, based on gene ontology, of the gene list for hypertension in the hypothalamus of the Schlager BPH/2J mouse.

| **Ontology** | **Representation** | **GOBPID** | **Term** | **Count** | **Adjusted *P* value** |
| --- | --- | --- | --- | --- | --- |
| BP | Over | GO:0007186 | G-protein coupled receptor protein signaling pathway | 297 | *P* <0.001 |
| BP | Over | GO:0006096 | glycolysis | 61 | *P* <0.001 |
| BP | Over | GO:0007608 | sensory perception of smell | 36 | *P* <0.001 |
| BP | Over | GO:0048739 | cardiac muscle fiber development | 15 | 0.002 |
| BP | Down | GO:0008150 | biological process | 6941 | *P* <0.001 |
| BP | Down | GO:0008015 | blood circulation | 31 | *P* <0.001 |
| BP | Down | GO:0030540 | female genitalia development | 29 | *P* <0.001 |
| BP | Down | GO:0043584 | nose development | 31 | *P* <0.001 |
| BP | Down | GO:0007628 | adult walking behavior | 54 | *P* <0.001 |
| BP | Down | GO:0016568 | chromatin modification | 229 | *P* <0.001 |
| BP | Down | GO:0042471 | ear morphogenesis | 31 | *P* <0.001 |
| BP | Down | GO:0006468 | protein amino acid phosphorylation | 451 | *P* <0.001 |
| BP | Down | GO:0007512 | adult heart development | 37 | *P* <0.001 |
| BP | Down | GO:0006333 | chromatin assembly or disassembly | 61 | *P* <0.001 |
| BP | Down | GO:0006350 | transcription | 1531 | *P* <0.001 |
| BP | Down | GO:0060021 | palate development | 55 | *P* <0.001 |
| BP | Down | GO:0040018 | positive regulation of multicellular organism growth | 47 | *P* <0.001 |
| BP | Down | GO:0007156 | homophilic cell adhesion | 62 | *P* <0.001 |
| BP | Down | GO:0007626 | locomotory behavior | 95 | *P* <0.001 |
| BP | Down | GO:0035116 | embryonic hindlimb morphogenesis | 50 | *P* <0.001 |
| BP | Down | GO:0006397 | mRNA processing | 291 | *P* <0.001 |
| BP | Down | GO:0007155 | cell adhesion | 342 | *P* <0.001 |
| CC | Over | GO:0005576 | extracellular region | 735 | *P* <0.001 |
| CC | Over | GO:0045095 | keratin filament | 11 | *P* <0.001 |
| CC | Over | GO:0042105 | alpha-beta T cell receptor complex | 7 | 0.002 |
| CC | Down | GO:0005575 | cellular_component | 6344 | *P* <0.001 |
| CC | Down | GO:0005634 | nucleus | 3733 | *P* <0.001 |
| CC | Down | GO:0016020 | membrane | 4326 | *P* <0.001 |
| CC | Down | GO:0005737 | cytoplasm | 3384 | *P* <0.001 |
| CC | Down | GO:0005622 | intracellular | 1375 | *P* <0.001 |
| CC | Down | GO:0005856 | cytoskeleton | 558 | *P* <0.001 |
| CC | Down | GO:0000785 | chromatin | 74 | *P* <0.001 |
| MF | Over | GO:0004984 | olfactory receptor activity | 23 | *P* <0.001 |
| MF | Over | GO:0030528 | transcription regulator activity | 259 | *P* <0.001 |
| MF | Over | GO:0043565 | sequence-specific DNA binding | 324 | *P* <0.001 |
| MF | Over | GO:0004930 | G-protein coupled receptor activity | 229 | *P* <0.001 |
| MF | Over | GO:0005179 | hormone activity | 39 | *P* <0.001 |
| MF | Over | GO:0003700 | transcription factor activity | 500 | *P* <0.001 |
| MF | Over | GO:0005125 | cytokine activity | 82 | 0.003 |
| MF | Down | GO:0046872 | metal ion binding | 2233 | *P* <0.001 |
| MF | Down | GO:0008270 | zinc ion binding | 1673 | *P* <0.001 |
| MF | Down | GO:0003674 | molecular_function | 6770 | *P* <0.001 |
| MF | Down | GO:0005524 | ATP binding | 1250 | *P* <0.001 |
| MF | Down | GO:0000166 | nucleotide binding | 1705 | *P* <0.001 |
| MF | Down | GO:0003676 | nucleic acid binding | 799 | *P* <0.001 |
| MF | Down | GO:0005515 | protein binding | 3547 | *P* <0.001 |
| MF | Down | GO:0004386 | helicase activity | 140 | *P* <0.001 |
| MF | Down | GO:0016787 | hydrolase activity | 1173 | *P* <0.001 |
| MF | Down | GO:0016740 | transferase activity | 1175 | *P* <0.001 |
| MF | Down | GO:0003682 | chromatin binding | 154 | *P* <0.001 |
| MF | Down | GO:0004672 | protein kinase activity | 416 | *P* <0.001 |
| MF | Down | GO:0005488 | binding | 693 | *P* <0.001 |
| MF | Down | GO:0004674 | protein serine/threonine kinase activity | 341 | *P* <0.001 |
| MF | Down | GO:0016301 | kinase activity | 626 | *P* <0.001 |
| MF | Down | GO:0003723 | RNA binding | 630 | *P* <0.001 |
| MF | Down | GO:0005509 | calcium ion binding | 602 | *P* <0.001 |
| MF | Down | GO:0008026 | ATP-dependent helicase activity | 65 | *P* <0.001 |
| MF | Down | GO:0004221 | ubiquitin thiolesterase activity | 58 | 0.001 |

*Ontology: BP, biological process; MF, molecular function; GOID: Gene Ontology identification. Shown is Term: ontology-associated term; Count: number of genes found that belong to ontology; the Benjamini-Hochberg false discovery rate-adjusted *P* value.
